# Tongkat Ali-Induced Atrial Flutter: A Probable Case

**DOI:** 10.7759/cureus.92024

**Published:** 2025-09-10

**Authors:** Mohamed Ali

**Affiliations:** 1 Cardiology, University Hospitals of Leicester, Leicester, GBR

**Keywords:** atrial flutter rapid ventricular response, herbal supplements, supra ventricular tachycardia, testosterone supplements, tongkat ali

## Abstract

We present the case of a 71-year-old gentleman who developed new-onset atrial flutter following the initiation of Tongkat Ali (Eurycoma longifolia) supplementation. He presented with a four-day history of palpitations and dizziness. Initial workup revealed normal laboratory values, including thyroid function and electrolytes. An electrocardiogram showed supraventricular tachycardia with a heart rate of 130 bpm, which, after adenosine administration, a clear atrial flutter. Secondary causes for atrial flutter were ruled out. Given the temporal association and the absence of other predisposing factors, his atrial flutter is presumed to be Tongkat Ali-induced, representing the first documented case in the literature. This case highlights the importance of considering herbal supplements as potential triggers for cardiac arrhythmias and underscores the need for increased awareness and further research into their cardiovascular safety profiles.

## Introduction

Approximately 18% of adults in the United States report using herbal supplements [1]. Tongkat Ali (Eurycoma longifolia), a traditional Southeast Asian herbal remedy, is commonly marketed for its purported benefits in enhancing libido, athletic performance, and testosterone levels [2]. While generally considered safe, the cardiovascular effects of herbal supplements are often underreported and poorly understood [3]. Atrial flutter is a common supraventricular arrhythmia characterized by rapid, regular atrial contractions at rates of 250-350 beats per minute, often presenting with a ventricular response of 150 beats per minute due to 2:1 atrioventricular conduction [4]. Although various medications and substances can trigger atrial flutter, herbal supplement-induced arrhythmias remain relatively uncommon in clinical practice [5]. We present a case of atrial flutter temporally associated with the initiation of Tongkat Ali supplementation in a previously healthy elderly male.

## Case presentation

**Figure 1:**
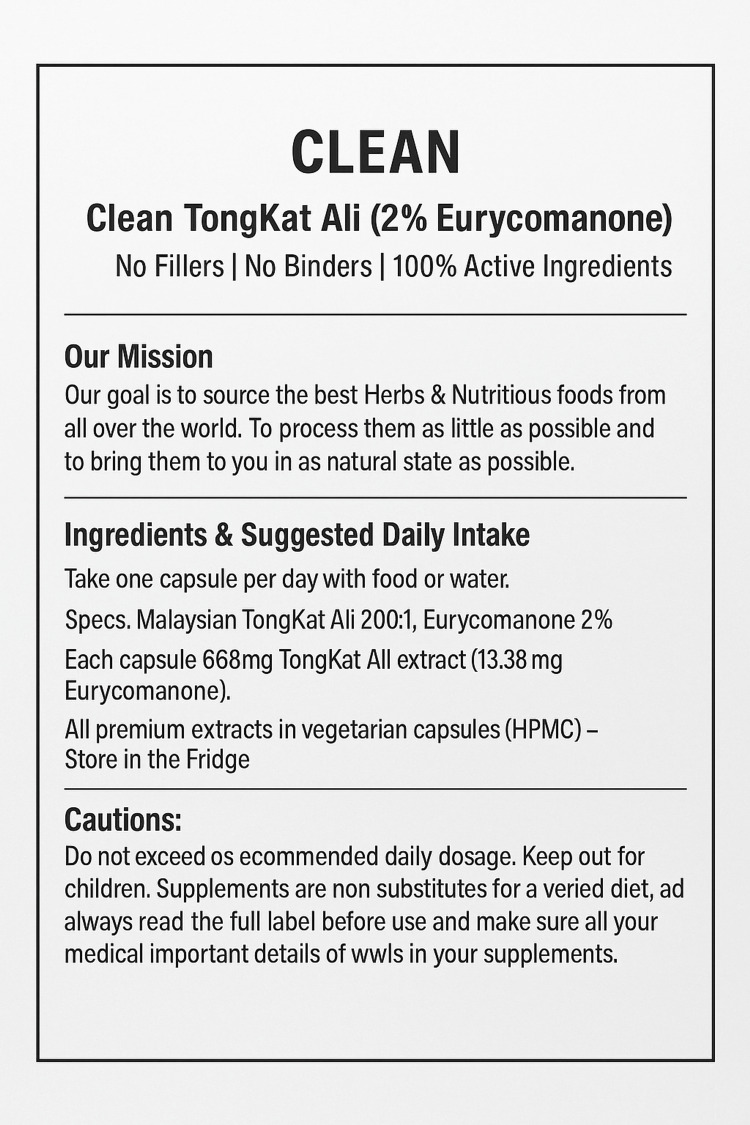
Tongkat Ali supplement label showing 668 mg extract per capsule with 2% eurycomanone content taken by the patient.

A 71-year-old gentleman sought emergency cardiovascular evaluation following a four-day history of palpitations and dizziness. He denied any episodes of syncope, chest pain, or shortness of breath. His medical history was notable for the absence of any significant comorbidities, and he maintained an active lifestyle, regularly exercising at the gym and reporting excellent health for his age. His social history was significant for being a lifelong non-smoker with minimal alcohol consumption and no history of excessive caffeine or stimulant use. Concurrently with the onset of his cardiovascular symptoms, he developed headache and nausea. During a comprehensive medical history, he disclosed that he had started taking a new herbal supplement, Tongkat Ali (668 mg capsule daily) (Figure 1), just two days before the onset of his symptoms.

On physical examination, his pulse was 145 bpm and regular, with a blood pressure of 95/55 mmHg. There were no signs of hypoperfusion or hemodynamic instability. Cardiovascular examination revealed a rapid but regular heart rhythm with no murmurs, gallops, or rubs. Pulmonary examination was unremarkable, and there were no signs of heart failure.

Initial laboratory investigations demonstrated normal findings across all parameters (Table 1).

**Table 1:** Laboratory investigations showing normal values across all parameters tested. eGFR: estimated glomerular filtration rate, TSH: thyroid-stimulating hormone

Laboratory Parameter	Patient Value	Units	Reference Range
Complete Blood Count			
Hemoglobin	158	g/L	130-180
Hematocrit	0.452	ratio	0.400-0.540
Platelets	234	×10⁹/L	140-400
White Blood Cell Count	7.4	×10⁹/L	4.0-11.0
Inflammatory Markers			
C-Reactive Protein	<5	mg/L	<5
Renal Function			
eGFR	72	mL/min/1.73m²	>60
Electrolytes			
Sodium	141	mmol/L	133-146
Potassium	4.2	mmol/L	3.5-5.5
Magnesium	0.90	mmol/L	0.7-1.0
Thyroid Function			
TSH	2.6	mIU/L	0.55-4.78
Free Thyroxine	14.0	pmol/L	11.0-22.0

**Figure 2:**
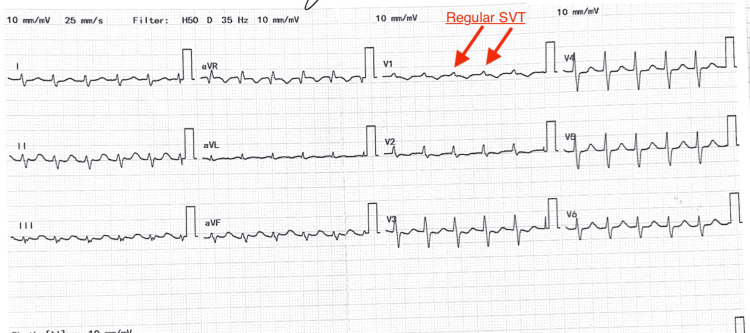
ECG showing supraventricular tachycardia (145 bpm, narrow QRS).

A 12-lead electrocardiogram revealed supraventricular tachycardia with narrow QRS complexes at a rate of 145 beats per minute as shown in Figure 2. 

**Figure 3:**
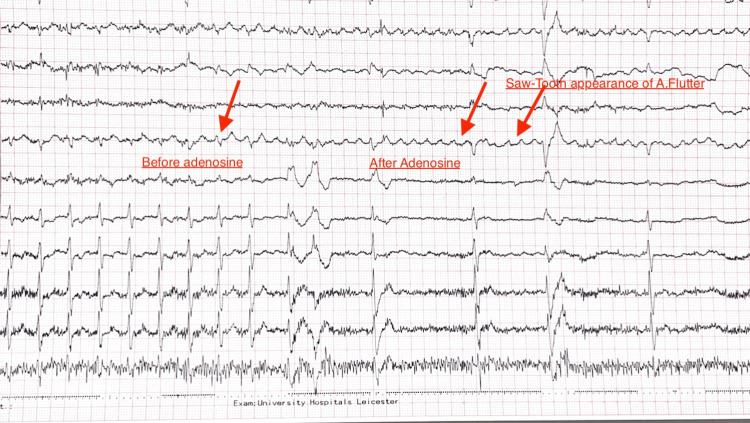
ECG Strip Before and After Adenosine Administration Revealing Atrial Flutter.

The patient's oxygen saturation was 98% on room air. Given the diagnostic uncertainty, intravenous adenosine was administered for both therapeutic and diagnostic purposes. An initial dose of 6 mg was given but failed to terminate the arrhythmia. A subsequent dose of 12 mg adenosine was administered, which successfully unmasked the underlying rhythm, revealing characteristic sawtooth flutter waves consistent with typical atrial flutter (Figure 3) [6].

**Figure 4:**
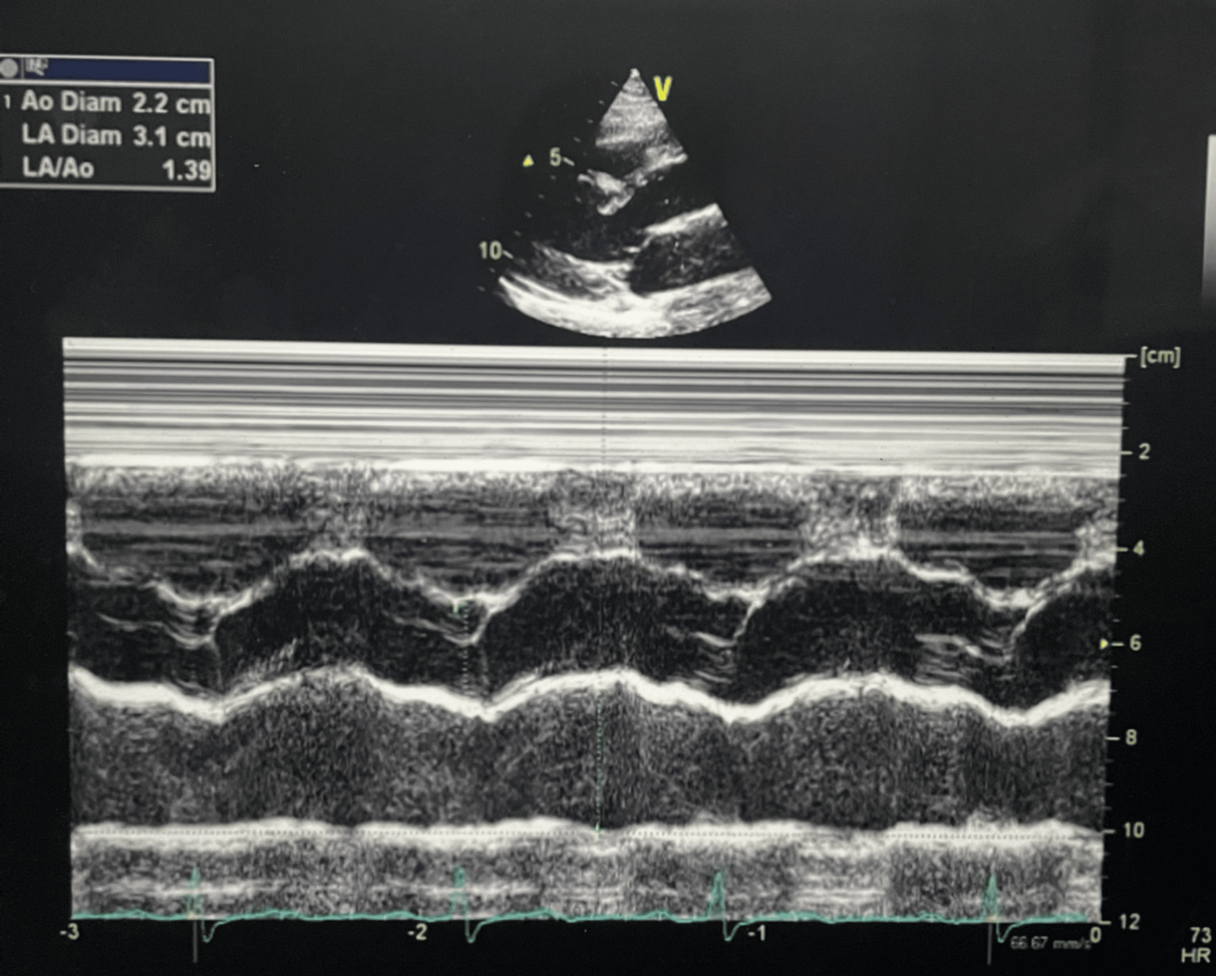
M-mode echocardiogram showing normal aortic root diameter (2.2 cm) and normal left atrial diameter (3.1 cm).

**Figure 5:**
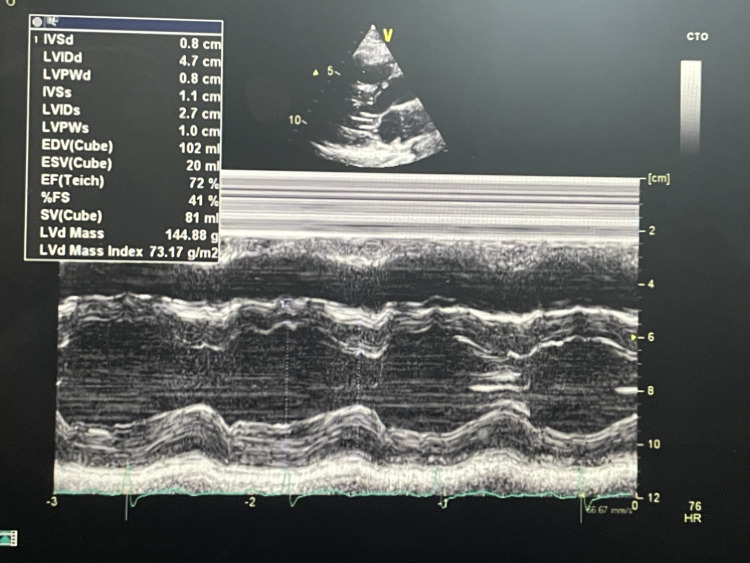
M-mode echocardiogram demonstrating normal left ventricular dimensions and normal function with ejection fraction of 72%.

Further investigations were performed to identify potential underlying causes of atrial flutter. Echocardiography demonstrated normal left ventricular function with ejection fraction of 72%, normal chamber dimensions, and no structural abnormalities (Figures 4, 5). All M-mode measurements within established reference ranges are shown in Table 2.

**Table 2:** M-Mode Echocardiography Normal Reference Values Abbreviations: LA = Left Atrium, Ao = Aortic Root, LVIDd = Left Ventricular Internal Diameter in Diastole, LVIDs = Left Ventricular Internal Diameter in Systole, IVSd = Interventricular Septal thickness in Diastole, LVPWd = Left Ventricular Posterior Wall thickness in Diastole, EF = Ejection Fraction, %FS = Fractional Shortening.

Parameter	Normal Range (Male)	Normal Range (Female)	Units
Aortic Root Dimensions			
Aortic Root Diameter	2.0-3.7	2.0-3.4	cm
Left Atrial Dimensions			
Left Atrial Diameter	2.3-4.3	2.3-3.9	cm
LA/Ao Ratio	0.9-1.4	0.9-1.4	ratio
Left Ventricular Dimensions			
LVIDd (Diastolic)	3.9-5.3	3.5-5.0	cm
LVIDs (Systolic)	2.1-4.0	2.0-3.5	cm
IVSd (Septal thickness)	0.6-1.0	0.6-0.9	cm
LVPWd (Posterior wall)	0.6-1.0	0.6-0.9	cm
Left Ventricular Function			
Ejection Fraction (EF)	≥55	≥55	%
Fractional Shortening (%FS)	≥27	≥27	%
Left Ventricular Mass			
LV Mass	88-224	67-162	g
LV Mass Index	49-115	43-95	g/m²
Stroke Volume	60-100	60-100	ml
End Diastolic Volume	96-157	76-130	ml
End Systolic Volume	22-58	19-49	ml

Given the temporal relationship between supplement initiation and symptom onset, combined with the absence of other identifiable triggers, the atrial flutter was attributed to Tongkat Ali supplementation. The supplement was immediately discontinued, and the patient was started on rate control therapy. The arrhythmia resolved within 24 hours of supplement cessation.

## Discussion

This case represents a rare but clinically significant adverse cardiovascular effect associated with Tongkat Ali supplementation. Eurycoma longifolia, commonly known as Tongkat Ali, is a flowering plant native to Southeast Asia that has been used traditionally for centuries as an aphrodisiac and general health tonic [7]. Modern marketing promotes its use for testosterone enhancement, improved athletic performance, and increased libido, leading to widespread availability as an over-the-counter supplement [8].

The mechanism by which Tongkat Ali might induce cardiac arrhythmias remains unclear. Proposed mechanisms include direct effects on cardiac ion channels, particularly sodium and potassium channels involved in cardiac conduction, or indirect effects through sympathetic nervous system activation [9]. Some studies suggest that Tongkat Ali may increase catecholamine levels, which could predispose to arrhythmogenesis [10]. Additionally, the supplement may contain various bioactive compounds that could have unpredictable cardiovascular effects, particularly in susceptible individuals [11]. Although challenge testing was not ethically feasible, resolution of arrhythmia within 24 hours of discontinuation strongly supports causality.

The temporal relationship in this case strongly suggests a causal association between Tongkat Ali initiation and atrial flutter development. The patient had no prior history of arrhythmias, structural heart disease, or other predisposing factors. The symptom onset occurred within 48 hours of supplement initiation, and the arrhythmia resolved after discontinuation. This temporal pattern is consistent with drug-induced arrhythmias and supports the likelihood of a causal relationship [12].

To formally assess the causal relationship between Tongkat Ali supplementation and atrial flutter, we applied the Naranjo Adverse Drug Reaction Probability Scale [13]. The assessment yielded a score of 6 points, indicating a probable causal relationship between Tongkat Ali use and the development of atrial flutter. Key contributing factors to this score included the clear temporal relationship between supplement initiation and arrhythmia onset (+2 points), complete resolution of symptoms upon discontinuation (+1 point), comprehensive exclusion of alternative etiologies through laboratory and imaging studies (+2 points), and objective confirmation of atrial flutter via electrocardiography and adenosine testing (+1 point). This standardized assessment provides objective evidence supporting our clinical observation and strengthens the case for Tongkat Ali as the probable causative agent in this patient's arrhythmia [13].

The diagnostic utility of adenosine in this case highlights its importance in differentiating supraventricular tachycardias. Adenosine acts by temporarily blocking atrioventricular nodal conduction, allowing underlying atrial activity to become visible on the electrocardiogram [14]. In atrial flutter, adenosine typically does not terminate the arrhythmia but reveals the characteristic flutter waves, as demonstrated in this case.

This case underscores several important clinical considerations. First, healthcare providers should routinely inquire about herbal supplement use during patient assessments, as patients may not spontaneously report such medications [15]. Second, the cardiovascular safety profiles of many herbal supplements remain poorly characterized, and adverse effects may be underrecognized or underreported [16]. Finally, the increasing popularity of herbal supplements necessitates greater awareness among clinicians regarding potential cardiovascular complications.

## Conclusions

We report the first case of Tongkat Ali-induced atrial flutter. This case highlights the potential for herbal supplements to cause significant cardiovascular adverse effects and emphasizes the importance of comprehensive medication histories that include over-the-counter and herbal products. Clinicians should maintain awareness of potential cardiovascular complications associated with herbal supplement use and consider them in the differential diagnosis of new-onset arrhythmias. Further research is needed to better characterize the cardiovascular safety profile of Tongkat Ali and other popular herbal supplements.
